# Multiple Mechanisms of Shenqi Pill in Treating Nonalcoholic Fatty Liver Disease Based on Network Pharmacology and Molecular Docking

**DOI:** 10.1155/2022/2384140

**Published:** 2022-06-26

**Authors:** Xiaojuan Tong, Sumei Xu, Dong Zhai

**Affiliations:** ^1^The First Affiliated Hospital of Zhejiang Chinese Medical University (Zhejiang Provincial Hospital of Traditional Chinese Medicine), Hangzhou 310003, China; ^2^The Third Affiliated Hospital of Zhejiang Chinese Medical University (Zhongshan Hospital of Zhejiang Province), Hangzhou 310005, China

## Abstract

**Background:**

Shenqi pill (SQP), a traditional Chinese prescription, has proven to be effective in treating nonalcoholic fatty liver disease (NAFLD). However, its bioactive ingredients and underlying mechanisms remain elusive.

**Aim:**

We aimed to predict the active compounds, potential targets, and molecular mechanisms of SQP anti-NAFLD by applying network pharmacology and molecular docking methods.

**Methods:**

Active ingredients and related targets of SQP were obtained from the TCMSP database. Potential targets of NAFLD were acquired from OMIM and GeneCards databases. The STRING database and Cytoscape software analyzed the protein-protein interaction (PPI) network and core targets of overlapping genes between SQP and NAFLD. GO enrichment analysis and KEGG enrichment analysis were performed in the DAVID database. Finally, molecular docking was employed to find possible binding conformations of macromolecular targets.

**Results:**

15 anti-NAFLD bioactive ingredients and 99 anti-NAFLD potential targets of SQP were determined using Network pharmacology. Quercetin, kaempferol, stigmasterol, diosgenin, and tetrahydroalstonine were the major active ingredients and AKT1, TNF, MAPK8, IL-6, and VEGFA were the key target proteins against NAFLD. The KEGG analysis suggested that the main pathways included PI3K/Akt signaling pathway, HIF-1 signaling pathway, MAPK signaling pathway, and TNF signaling pathway. Molecular docking predicted that quercetin, kaempferol, stigmasterol, diosgenin, and tetrahydroalstonine could bind with AKT1, TNF, and MAPK8.

**Conclusion:**

This study successfully predicts the active compounds, potential targets, and signaling pathways of SQP against NAFLD. Moreover, this study contributed to the application and development of SQP.

## 1. Introduction

Nonalcoholic fatty liver disease (NAFLD) is characterized by excessive fat accumulation in liver cells and has been recognized as a leading cause of chronic liver disease worldwide [1, 2]. NAFLD progress to nonalcoholic steatohepatitis (NASH), fibrosis, cirrhosis, and even hepatocellular carcinoma [3]. NAFLD not only increases the risk of diabetes, metabolic syndrome, and cardiovascular and cerebrovascular diseases but also is closely related to the high incidence of chronic diseases such as osteoporosis, chronic kidney disease, colorectal cancer, and breast cancer [4–9]. The global prevalence of NAFLD is estimated at 25% [10]. Meanwhile, the incidence of NAFLD in children increased dramatically in recent years [11]. However, there is still a lack of useful drugs for NAFLD treatment, so finding and developing new effective drugs is necessary.

Shenqi pill (SQP) is a famous traditional Chinese medicine (TCM) prescription for treating various liver and renal diseases in China. It was first described in the book named JinGuiYaoLue written by Zhongjing Zhang. The formula consists of eight herbs: Rehmannia Glutinosa (Shu Di Huang: SDH), Chinese Yam (Shan Yao: SY), Cornus Officinalis (Shan Zhu Yu: SZY), Alisma Orientalis (Fu Ling: FL), Poria (Ze Xie: ZX), Moutan Bark (Mu Dan Pi: MDP), Cassia Twig (Gui Zhi: GZ), and Aconite (Fu Zi: FZ). Previous studies found that SQP has a series of effects, such as ameliorating renal fibrosis [12], regulating immunity [13], and promoting memory function [14, 15]. Research has also shown that SQP is a safe and effective formula for treating NAFLD. Wu et al. showed that SQP improved the levels of aspartate aminotransferase (AST), alanine aminotransferase (ALT), total cholesterol (TC), and low-density lipoprotein(LDL) in rats with steatohepatitis by regulating Bcl-2/Bax and Fas/FasL signaling pathways [16]. However, its underlying mechanisms require in-depth exploration.

Network pharmacology is a promising tool to identify the scientific basis and mechanism of TCM at the systemic level [17–19]. We used network pharmacology to predict the active compounds, potential targets and signaling pathways of SQP against NAFLD. Moreover, we performed molecular docking studies to predict possible binding conformations of macromolecular targets.

## 2. Materials and Methods

### 2.1. Screening Active Ingredients and Target Proteins of SQP

All bioactive compounds of eight herbs in SQP were searched from the Traditional Chinese Medicine Systems Pharmacology Database and Analysis Platform (TCMSP, http://tcmspw.com/tcmsp.php) [20]. ADME criteria, including absorption, distribution, metabolism, and excretion, were adopted to choose bioactive ingredients. Oral bioavailability (OB) is one of the important pharmacokinetic parameters in ADME, showing the ratio of the drug absorbed by the body [21]. Drug-likeness (DL) represents the similarity of its ingredients compared with known chemical drugs [22]. The higher the OB value, the better the DL. We selected active compounds with OB ≥ 30% and DL ≥ 0.18 for further research. We extracted the corresponding protein targets of SQP from the TCMSP database and transformed them into their related potential gene symbols via UniProt KB (https://www.uniprot.org/).

### 2.2. Prediction Gene Targets of NAFLD

The related targets of nonalcoholic fatty liver disease (NAFLD) were acquired from two major databases: OMIM (https://www.omim.org/) [23] and GeneCards Database (https://www.genecard.org/) [24].

### 2.3. Construction of Herb-Active Compound-Disease-Target Interaction Network

In the beginning, we used venny2.1 to obtain overlapping genes of SQP and NAFLD as hub genes. Subsequently, an herb-active compound-disease-target interaction network (C-D-T) of treatment with SQP against NAFLD was built by Cytoscape 3.7.2.

### 2.4. PPI Network Construction

We imported the hub targets into the STRING database (https://www.string-db.org/) to construct the protein-protein interaction (PPI) network. The species was set to “*Homo sapiens*” and an interaction score >0.7. Finally, the TSV format file was input into Cytoscape 3.7.2 for graphical visualization.

### 2.5. Enrichment Analysis

Based on the DAVID database (https://david.ncifcrf.gov/), the Gene Ontology (GO) enrichment analysis, and Kyoto Encyclopedia of Genes and Genomes (KEGG) enrichment analysis of overlapping target proteins were obtained. GO enrichment analysis was applied to show the functions of gene targets, including three parts: biological process (BP), molecular function (MF), and cellular component (CC). The KEGG enrichment analysis described the distribution of hub targets in relevant pathways. In this study, we set *p* < 0.05 as statistically significant. We select the top 10 most enriched BP, MF, CC, and pathways to draw bar charts and bubble plots by using the WeChat online software.

### 2.6. Molecular Docking

We applied AutoDockTools-vina to perform molecular docking to reveal the interaction between active ingredients (ligands) and target proteins (receptors). We downloaded the 2D structures of the compounds from the PubChem Database and used Chem3D software to transform them into 3D structures with minimizing energy. The 3D structure of proteins was downloaded from the Protein Data Bank (PDB, http://www.rcsb.org/). The PyMOL software was used to dehydrate and remove ligand residues of receptor proteins. The receptor protein was hydrogenated using the AutoDockTool 1.5.6 software and saved in the pbdqt format. The ligand was also saved in the pdbqt format. The active pocket site was built to cover the entire protein. Finally, we used AutoDock Vina for docking and looked for the optimal conformation. A total of 20 conformations were generated for each ligand-protein docking study. The lower the docking score, the more stable the binding between the protein and the molecule. The best scoring conformer with a minimum energy of a drug molecule and target was visualized in PyMOL.

## 3. Results

### 3.1. Active Ingredients of SQP

A total of 102 active ingredients of SQP were retrieved from the TCMSP database that satisfied the criteria of OB ≥ 30% and DL ≥ 0.18, including 2 kinds in Shudihuang, 16 kinds in Shanyao, 20 kinds in Shanzhuyu, 15 kinds in Fuling, 10 kinds in Zexie, 11 kinds in Mudanpi, 7 species in Guizhi, and 21 species in Fuzi. After eliminating 10 duplicates, there were 92 ingredients for further study.

### 3.2. Potential Target Genes of SQP and NAFLD

**Figure 1:**
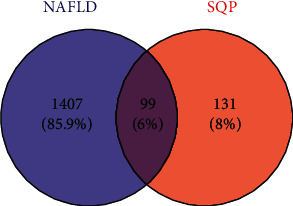
Venn diagram of targets of nonalcoholic fatty liver disease (NAFLD) and Shenqi pill (SQP).

A total of 231 active ingredient targets of SQP were selected from the TCMSP database. Meanwhile, a total of 1508 related targets for NAFLD were acquired using GeneCards and OMIM databases. Among the 231 ingredient targets and 1508 NAFLD related target genes, we acquired 99 intersection genes through the venny2.1 software, which are the potential target of SQP in treating NAFLD. Then, we found the compounds corresponding to 99 intersecting genes and deleted the repetitions. Finally, we obtained 15 interaction target-related compounds. Degree represented the total number of gene targets corresponding to this compound. The details are shown in Figure 1 and Table 1. Quercetin, kaempferol, and stigmasterol are the top 3 degree compounds.

### 3.3. Construction of Herb-Active Compound-Disease-Target Interaction (C-D-T) Network

**Figure 2:**
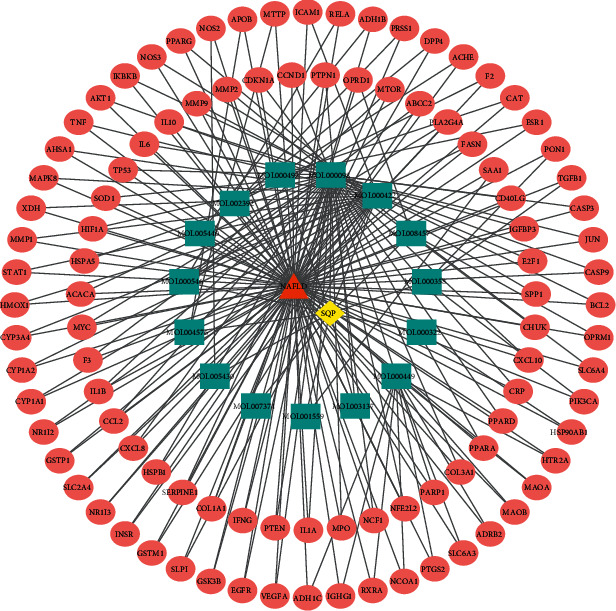
Compounds and corresponding target network of SQP with NAFLD. The blue rectangle stands for active ingredients, the red ellipse node represents the potential targets of active compounds, the yellow diamond represents SQP, and the orange triangle represents NAFLD.

We imported 99 corresponding targets, 15 bioactive ingredients, SQP, and NAFLD into the Cytoscape3.7.2 software to construct the (C-D-T) networks. As shown in Figure 2, it contains 116 nodes and 213 edges. The blue rectangle stands for active ingredients, the red ellipse node represents the potential targets of active compounds, the yellow diamond represents SQP, and the orange triangle represents NAFLD. The connections between nodes are edges, representing the degree of association between the active ingredients and the targets. The higher the degree value, the more important the nodes in the network. Quercetin, kaempferol, and stigmasterol have the highest degree, suggesting that they play major roles in the effect of SQP anti-NAFLD.

### 3.4. Construction and Analysis of PPI Network

**Figure 3:**
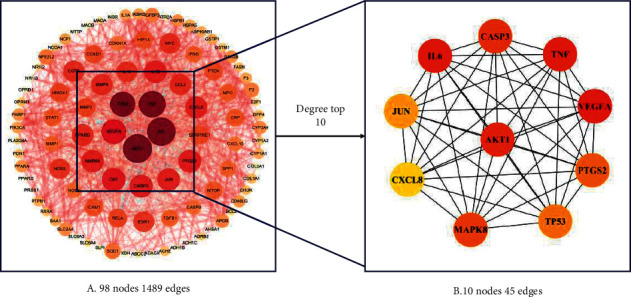
Identification of potential targets of Shenqi pill on NAFLD via PPI analysis. (a) PPI network of hub genes between SQP and NAFLD via STRING database and Cytoscape. (b) The top 10 degree targets of overlapping targets.

The PPI networks were constructed by importing 99 overlapping targets into the STRING database and then visualized in the Cytoscape software. As shown in Figure 3(a), the network contains 98 nodes and 1489 edges. Yellow stands for the lowest degree, and red represents the highest degree. The larger the node size, the higher the degree. The top 10 targets with the highest degree value were AKT1, TNF, MAPK8, IL6, TP53, JUN, CASP3, CXCL8, VEGFA, and PTGS2, as shown in Figure 3(b). They play a critical role in the PPI network for the SQP with NAFLD.

### 3.5. Gene Ontology Enrichment Analysis

**Figure 4:**
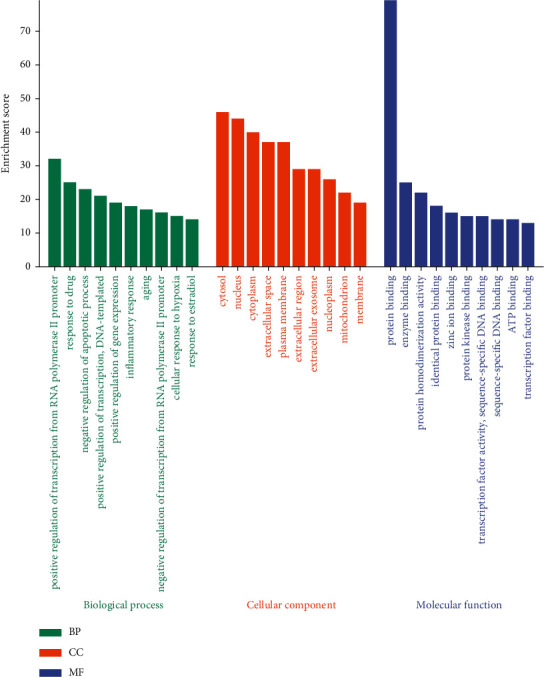
Gene ontology enrichment analysis. The top 10 significantly enriched biological processes (BP), cellular component (CC), and molecular function (MF) are shown in green, orange, and purple bars, respectively.

GO enrichment analysis was constructed by the DAVID database. In total, 655 GO terms meet the demand *p* < 0.05, including 508 biological processes (BP), 55 cellular components (CC), and 92 molecular functions (MF). We selected the top 10 terms from BP, CC, and MF, respectively, based on the −log10 (*p* value), as shown in Figure 4. The most significantly enriched BP, CC, and MF were positive regulation of transcription from RNA polymerase II promoter, cytosol, and protein binding, respectively.

### 3.6. KEGG Enrichment and Pathway-Target (P-T) Network Analysis

**Figure 5:**
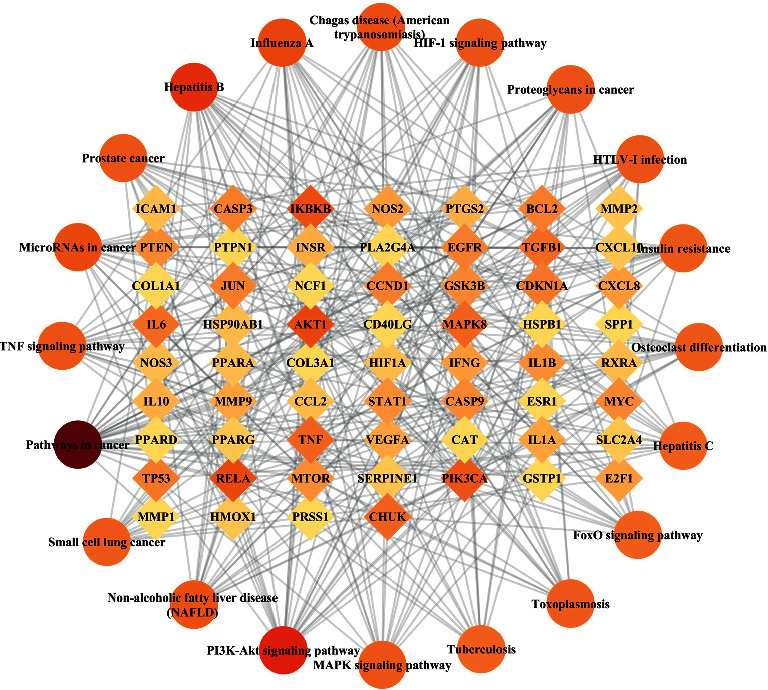
Pathway-target network of top 20 pathways. The diamonds represent genes, the circles stand for pathways, red denotes the high degree value, and yellow represents the low degree value.

**Figure 6:**
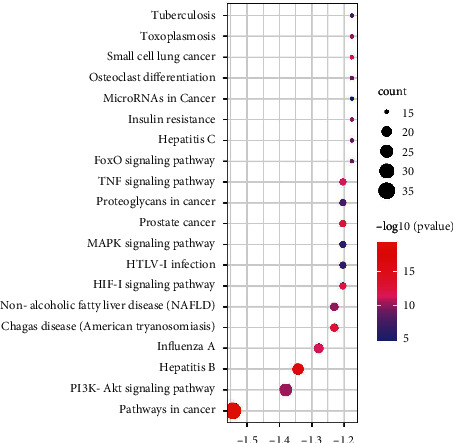
The top 20 significantly enriched signal pathways from KEGG analysis.

KEGG enrichment analysis indicated that 111 terms were related to liver disease, including cancer pathway, PI3K/Akt signaling pathway, non-alcoholic fatty liver disease (NAFLD), MAPK signaling pathway, HIF-1 signaling pathway, and TNF signaling pathway. We selected the top 20 entries as core pathways based on −log10 (*p* value), as shown in Figure 5. The cancer pathway and PI3K/Akt signaling pathway are two major signaling pathways for treating the Shenqi pill with NAFLD. The pathway-target networks demonstrate the interactions of overlapping targets and the top 20 pathways. As shown in Figure 6, the diamonds represent genes, the circles stand for pathways. Red denotes the high degree value, and yellow represents the low degree value.

### 3.7. Molecular Docking Analysis

**Figure 7:**
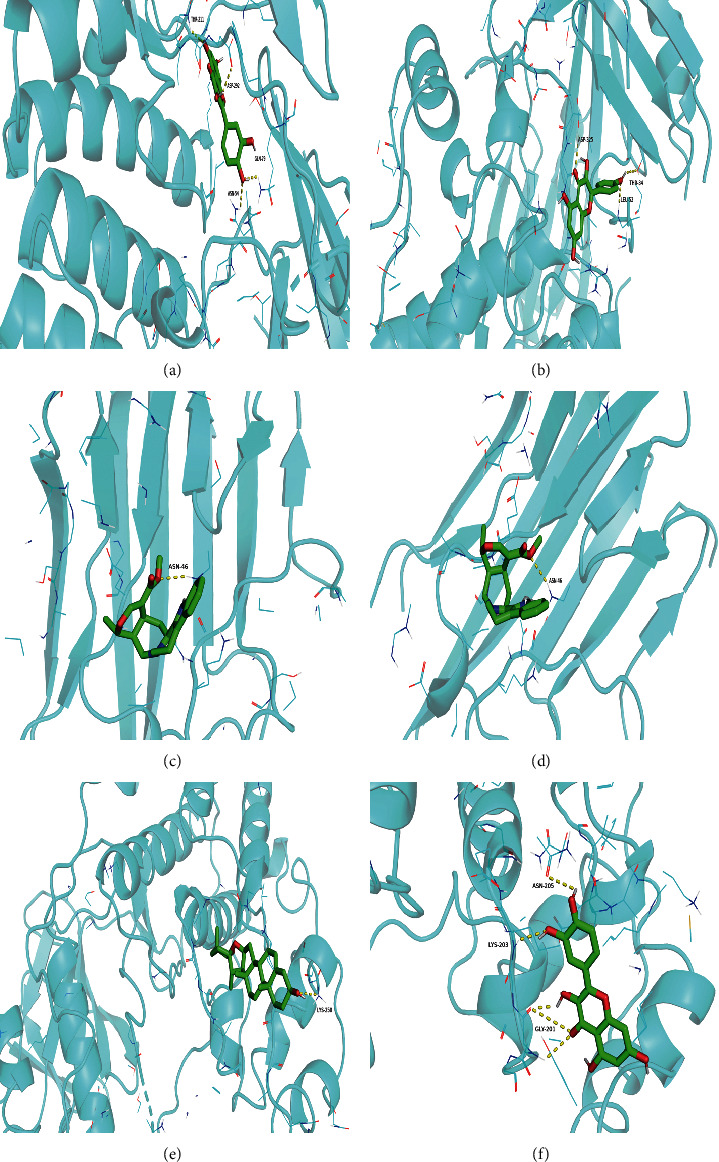
(a–f) Representative results of molecular docking. (a) Quercetin-AKT1; (b) kaempferol-AKT1; (c) kaempferol-TNF; (d) tetrahydroalstonine-TNF; (e) diosgenin-MAPK8; (f) quercetin-MAPK8.The ingredient structure was shown as a green stick, the protein structure was presented as a blue ribbon, and the hydrogen bonds were shown as a yellow chain.

The top five active ingredients with the highest degree (quercetin, kaempferol, stigmasterol, diosgenin, and tetrahydroalstonine) and three key target genes (AKT1, TNF, and MAPK8) were selected to perform molecular docking. The docking energy score is listed in Table 2, the lower the energy, the more stable the structure. The results have shown that the docking scores of quercetin to AKT1, kaempferol to TNF, diosgenin to MAPK8 were −9.4 kcal/mol, −6.4 kcal/mol, and −8.8 kcal/mol, respectively. As shown in Figures 7(a)–7(f), quercetin is bound to AKT1 with 4 hydrogen bonds: THR-211, ASP-292, GLN-79, and ASN-54. Kaempferol was attracted to TNF by ASN-46 hydrogen. When diosgenin encountered MAPK8, it formed only 1 hydrogen bond: LYS-250.

## 4. Discussion

In this study, network pharmacology and molecular docking method were employed to clarify the active compounds and molecular mechanism of SQP for NAFLD treatment. A total of 99 overlapping target genes and 15 active compounds were selected for SQP against NAFLD. The top 5 highest degree ingredients include quercetin, kaempferol, stigmasterol, diosgenin, and tetrahydroalstonine, as shown in Table 1. Yang et al. revealed that quercetin improved non-alcoholic fatty liver by ameliorating inflammation, oxidative stress, and lipid metabolism in db/db mice [25]. Furthermore, the current study also shows that quercetin improves glycolipid metabolism disorder by regulating the SIRT1 protein and AKT signaling pathway [26]. Stigmasterol and *β*-sitosterol can regulate the expression of lipid metabolism genes, thus improving lipid metabolism and reducing the level of bile acid in the intestine [27, 28]. The above results suggest that the active ingredients of the Shenqi pill can improve NAFLD.

PPI network shows AKT1, TNF, MAPK8, IL6, TP53, JUN, CASP3, CXCL8, VEGFA, PTGS2, especially AKT1, play a major effect on SQP anti-NAFLD. By GO and KEGG enrichment analysis, we found that the major signaling pathways related to NAFLD were pathways in cancer, PI3K-Akt signaling pathway, and HIF-1 signaling pathway. A recent study has found that the activation of the PI3K-Akt signaling pathway could inhibit the expression of SREBP1c and PPAR*α* protein, finally resulting in lipid metabolism disorders and insulin resistance, promoting the process of NAFLD [29]. Hypoxia-inducible factor 1 (HIF-1), as an oxygen-sensing transcription factor, is well known to take a major participant in the control of metabolisms, such as nonalcoholic fatty liver disease, type 2 diabetes mellitus, and obesity [30]. A previous study has shown that activating HIF-1 can promote liver fibrosis in the liver cell [31].

Furthermore, molecular docking was applied to simulate the binding ability of different compounds and proteins. Compared with other compounds, the binding energy of quercetin to AKT1 was the lowest, which was about −9.4 kcal/mol. The lower the energy score, the stronger the binding capacity. Similarly, the combined energy of kaempferol-TNF was −6.4 kcal/mol and that of diosgenin-MAPK8 was −8.8 kcal/mol. These results strongly indicate that the active ingredients of SQP can effectively treat NAFLD through major binding genes. Huang et al. [17] also found that quercetin interacts with AKT1 through a hydrogen bond. Therefore, AKT1 may be the key target of quercetin's anti-colorectal cancer effect. A study by Huang et al. reveals that quercetin well matches MAPK8 [32]. A previous study confirmed that kaempferol is the effective ingredient of Tripterygii Radix anti-RA-FLS and its involvement in regulating AKT1, TNFR1, TNFR2, and TNF-*α* expression [33].

This study reveals the theoretical molecular mechanism of the ingredients of SQP for the treatment of NAFLD through network pharmacology and verified by molecular docking. However, further pharmacological and clinical studies are needed to validate the therapeutic mechanism of SQP.

## 5. Conclusions

The present study demonstrated the active compounds, potential genes, and signal pathways of SQP in treating NAFLD based on network pharmacology. We found that quercetin, kaempferol, stigmasterol, diosgenin, and tetrahydroalstonine are the main active ingredients of SQP. The core target genes of SQP for the treatment are AKT1, TNF, and MAPK8. SQP plays a major effect in NAFLD treatment by regulating the PI3K-Akt signaling pathway, TNF signaling pathway, and MAPK signaling pathway. Overall, this study provides a promising and scientific basis for further investigation of SQP for NAFLD treatment.

## Figures and Tables

**Table 1:** Active compounds of SQP against NAFLD.

Mol ID	Ingredient	Degree	OB (%)	DL
MOL000098	Quercetin	41	54.83	0.24
MOL000422	Kaempferol	22	65.31	0.35
MOL000449	Stigmasterol	10	43.87	0.76
MOL000546	Diosgenin	5	43.78	0.76
MOL008457	Tetrahydroalstonine	4	32.42	0.81
MOL002392	Deltoin	4	46.69	0.37
MOL000358	Beta-sitosterol	3	36.91	0.75
MOL000492	(+)-Catechin	2	42.36	0.37
MOL004576	Taxifolin	2	57.84	0.27
MOL000322	Kadsurenone	1	37.57	0.71
MOL005440	Isofucosterol	1	38.16	0.54
MOL005430	Hancinone C	1	61.47	0 38
MOL007374	5- ((5- (4-methoxyphenyl)-2furyl)methylene)Barbiturc acid	1	31.14	0.54
MOL001559	Piperlonguminine	1	60.51	0.27
MOL003137	Leucanthoside MOL005440	1	32.12	0.78

**Table 2:** The binding energy and interactions of ingredients bound to key targets.

ID	Ingredient	Bind energy (kcal/mol)		
		AKT1	TNF	MAPK8
MOL000098	Quercetin	−9.4	−6.1	−7.5
MOL000422	Kaempferol	−7.1	−6.4	−7.4
MOL000449	Stigmasterol	−5.7	−5.3	−7.2
MOL000546	Diosgenin	−7.1	−5.4	−8.8
MOL008457	Tetrahydroalstonine	−6.4	−6.3	−7.0

## Data Availability

All the data used in this study are shown in figures and tables.
